# Randomized placebo-controlled trial of high-dose prenatal third-trimester vitamin D3 supplementation in Bangladesh: the AViDD trial

**DOI:** 10.1186/1475-2891-12-47

**Published:** 2013-04-12

**Authors:** Daniel E Roth, Abdullah Al Mahmud, Rubhana Raqib, Evana Akhtar, Nandita Perumal, Brendon Pezzack, Abdullah H Baqui

**Affiliations:** 1 Department of Paediatrics, Hospital for Sick Children and University of Toronto, 555 University Avenue, Toronto, ON M5G 1X8, Canada; 2 International Center for Diarrhoeal Disease Research, Bangladesh (icddr, b), GPO Box 128, Dhaka 1000, Bangladesh; 3 Department of International Health, The Johns Hopkins Bloomberg School of Public Health, 615 North Wolfe Street, Baltimore, MD 21205, USA

**Keywords:** Vitamin D, Bangladesh, Pregnancy, Pharmacokinetics, Hypercalcemia

## Abstract

**Background:**

Antenatal vitamin D status may be associated with the risk of adverse pregnancy and neonatal outcomes; however, the benefits of vitamin D supplementation during pregnancy remain unknown.

**Methods:**

We conducted a double-blind placebo-controlled randomized trial to evaluate the effect of high-dose prenatal 3^rd^ trimester vitamin D3 supplementation on maternal and neonatal (cord blood) serum 25-hydroxyvitamin D (25(OH)D) concentration (primary biochemical efficacy outcome) and maternal serum calcium concentration (primary safety measure). Eligibility criteria were pregnant women aged 18 to <35 years, at 26 to 29 weeks gestation, and residing in Dhaka, Bangladesh. 160 women were randomized by 1:1 allocation to one of two parallel intervention groups; placebo (n = 80) or 35,000 IU/week of vitamin D3 (n = 80) until delivery. All participants, study personnel and study investigators were blind to treatment allocation.

**Results:**

Mean maternal 25(OH)D concentration was similar in the vitamin D and placebo groups at baseline (45 vs. 44 nmol/L; p = 0.66), but was significantly higher in the vitamin D group vs. placebo group among mothers at delivery (134 vs. 38 nmol/L; p < 0.001) and newborns (cord blood: 103 vs. 39; p < 0.001). In the vitamin D group, 95% of neonates and 100% of mothers attained 25(OH)D >50 nmol/L, versus 21% mothers and 19% of neonates in the placebo group. No participants met criteria for hypercalcemia, there were no known supplement-related adverse events, and major pregnancy outcomes were similar between groups.

**Conclusions:**

Antenatal 3^rd^-trimester vitamin D3 supplementation (35,000 IU/week) significantly raised maternal and cord serum 25(OH)D concentrations above 50 nmol/L in almost all participants without inducing hypercalcemia or other observed safety concerns. Doses up to 35,000 IU/week may be cautiously used in further research aimed at establishing the clinical effects and safety of vitamin D3 supplementation in pregnancy.

**Trial registration:**

This trial was registered at ClinicalTrials.gov (NCT01126528).

## Introduction

Vitamin D status during pregnancy has been proposed to influence the risk of gestational diabetes and hypertensive diseases of pregnancy [1], fetal skeletal growth [2], brain development [3], and neonatal immune function [4]. The major circulating vitamin D metabolite, 25-hydroxyvitamin D (25(OH)D), crosses the placenta from the maternal to the fetal circulation, thereby establishing fetal-neonatal vitamin D stores [5]. However, the effects of vitamin D on maternal, placental or fetal tissues are uncertain [2], and the extent to which health outcomes are responsive to changes in maternal-fetal vitamin D status remains unknown [2,6,7].

Currently, there is insufficient evidence to either support or refute the benefits of any given supplemental vitamin D dose during pregnancy [8,9]. Moreover, the safety of high-dose prenatal vitamin D regimens (i.e., doses that substantially exceed the conventional prenatal intake of 400 to 600 IU/day) has yet to be established [6]. The Institute of Medicine (IOM) Committee to Review Dietary Reference Intakes (DRIs) for Vitamin D and Calcium concluded in 2010 that the recommended dietary allowance (RDA) of 600 IU/day and tolerable upper intake level (UL) of 4000 IU/day for non-pregnant adults were suitable for pregnant women [7]. The UL was doubled from 2000 IU/day, as recommended by the IOM in 1997 [10], despite a lack of new pregnancy trial safety data published between 1997 and 2010. Uncertainty surrounding vitamin D requirements in pregnancy has led to divergent dose recommendations; for example, the UNICEF antenatal micronutrient formulation contains 200 IU/day [11], the Canadian Paediatric Society has suggested 2000 IU/day [12], and Hollis et al. advised an intake of 4000 IU/day [13]. Similarly, the 25(OH)D threshold to define vitamin D sufficiency is debated; the IOM set 50 nmol/L as a lower limit of sufficiency [7], yet other expert bodies such as the American Academy of Pediatrics have suggested that pregnant women attain serum 25(OH)D >80 nmol/L [14].

Determination of the vitamin D intake level (and corresponding serum 25(OH)D concentration) that safely optimizes vitamin D-responsive maternal-infant health outcomes would have global implications, but may be particularly relevant to resource-poor communities in South Asia, where a relatively low prenatal vitamin D status overlaps with a high burden of morbidity and mortality associated with adverse birth and early infant health outcomes [15]. In open-label pilot studies of high-dose vitamin supplementation in non-pregnant and pregnant women in Dhaka, Bangladesh, we showed that 35,000 IU/week was well tolerated and raised 25(OH)D to >80 nmol/L in pregnant women and newborns [16]. To more rigorously establish the biochemical effects of this regimen, and to provide evidence for a UL in pregnancy, we conducted a randomized controlled trial (RCT) of 3^rd^-trimester vitamin D3 supplementation of 35,000 IU per week in Dhaka. The primary aim of the study was to evaluate the change in maternal and neonatal (cord blood) 25(OH)D concentration and effects on serum calcium concentration, and to generate preliminary data regarding pregnancy outcomes. These data may serve to design larger RCTs to measure the effects of antenatal vitamin D supplementation on functional outcomes of clinical and public health importance.

## Subjects and methods

### Study design and participants

The Antenatal Vitamin D in Dhaka (AViDD) trial was a double-blind placebo-controlled RCT conducted in Dhaka, Bangladesh (23°N), through collaboration of the International Center for Diarrheal Disease Research, Bangladesh (icddr,b), The Johns Hopkins Bloomberg School of Public Health (Baltimore), and the Hospital for Sick Children (Toronto). Enrolment of 160 participants occurred from August 2010 to January 2011, and pregnancies were completed between September 2010 and April 2011. Participants were enrolled at the Shimantik Urban Primary Health Care Project maternity center, a non-governmental, non-profit facility that provides basic antenatal and obstetric services (including cesarean deliveries) in a low-income area of Dhaka (Khilgaon division). Participants were screened for eligibility when they presented to the clinic for antenatal care (ANC) visits during regular daytime clinic hours, and were offered enrolment if they met the following inclusion criteria: aged 18 to <35 years; gestational age of 26 to <30 weeks, estimated based on the first day of the last menstrual period (LMP); current residence in Dhaka at a fixed address; planned to deliver at the Shimantik maternity center, and to stay in Dhaka throughout the pregnancy and for at least one month past the date of delivery. Individuals were not eligible if they had any of the following exclusion criteria: use of any dietary supplement containing more than 400 IU/day (10 mcg/day) of vitamin D within the month prior to enrolment, or refusal to stop taking supplemental vitamin D at any dose after enrollment; current use of anti-convulsant or anti-mycobacterial (tuberculosis) medications; severe anemia (hemoglobin < 70 g/L); systolic blood pressure ≥140 mm Hg or diastolic blood pressure ≥90 mm Hg; positive urine dipstick for proteinuria or glycosuria; complicated medical or obstetric history; or, reported prior history of delivery of an infant with a major congenital anomaly, birth asphyxia, or perinatal death. Informed consent was obtained from all participants. The study was approved by ethical review boards at The Johns Hopkins Bloomberg School of Public Health, Hospital for Sick Children, and icddr,b. The trial was registered at ClinicalTrials.gov (NCT01126528).

### Intervention

Participants were randomly assigned to one of two masked parallel intervention groups, with allocation concealment: vitamin D3 (cholecalciferol) 35,000 IU/week or matched placebo. The vitamin D3 was a high-concentration (20,000 IU D3 per mL) liquid formulation (Vigantol Oil, Merck KGaA, Germany), and the placebo was miglyol oil 812 (Sasol, Germany), the vehicle used in Vigantol Oil. Assignment was based on a computer-generated randomization list, with 1:1 allocation, using permuted blocks of size 4 and 8. The allocation sequence was prepared by icddr,b personnel not otherwise involved in the study, and was concealed from investigators. Popular Pharmaceuticals Ltd. (Dhaka) prepared the supplements off-site using individual opaque glass vials labeled with unique participant identifiers based on the randomization list. Vials were maintained at ambient temperature at the clinic throughout the study. The active and placebo supplements were identical in appearance and tasteless. Participants and research staff (including lab personnel) were blinded to allocation. Supplement doses were measured in disposable plastic syringes and orally administered by study personnel, beginning at the baseline visit and continuing thereafter at 7-day intervals until delivery. Missed doses could be administered up to 7 days after the scheduled date, but otherwise were skipped. Participants were advised to discontinue all other supplements containing vitamin D, and were offered prenatal iron and folic acid supplements (66 mg elemental iron and 350 mcg/day folic acid per day), routine antenatal monitoring, and obstetric care free of charge.

### Data collection and laboratory analyses

The baseline visit consisted of a detailed questionnaire, anthropometry, blood pressure measurement, specimen collection, and supplement administration. Thereafter, participants were contacted weekly at their homes or in the clinic for supplement administration and a questionnaire that included a checklist of symptoms related to pregnancy complications and hypo-/hypercalcemia. Random spot urine specimens were collected at baseline, 2 weeks post-enrolment, and delivery. Venous blood specimens were collected at baseline and once between gestational weeks 30 and 37 (the specific week was randomly varied among participants); these specimens were drawn immediately preceding the weekly vitamin D dose administration; a third specimen was collected around the time of delivery, irrespective of when the last vitamin D dose was received. Study personnel were on-call to attend deliveries at the maternity center, where they completed a delivery record and collected maternal and cord blood specimens. A physician examined all infants within 2 days of birth. Newborn weight was measured with a digital scale (Seca 354, Seca Corporation, Hanover, USA), length was measured using a locally-manufactured wooden length board. Anthropometric results were the averages of paired repeated measures.

Maternal venous blood was collected into serum separator tubes by standard methods, and cord blood specimens were collected from the umbilical vein immediately after delivery of the placenta. Maternal and cord serum samples were maintained at 4°C prior to same-day transfer to the laboratory. Serum aliquots were frozen at −20°C and shipped at ambient temperature from Bangladesh to Toronto for measurement of serum 25(OH)D concentration, a well-established biomarker of systemic vitamin D status [17]. Serum 25(OH)D concentrations are unaffected by serum storage for several days at refrigerated or room temperatures [18,19], and 25(OH)D is resistant to multiple freeze-thaw cycles [18,20]. Serum 25(OH)D was quantified by high-performance liquid chromatography tandem mass spectroscopy (LC-MS/MS) in the Department of Pathology and Laboratory Medicine at the Hospital for Sick Children. Briefly, internal standards (50 μL, 10 μmol/L) were added to 200 μL of serum, calibrator and control samples. Vitamin D metabolites were extracted from the samples with 4 mL of hexane/methanol (3:1). The excess solvent was evaporated to dryness under a stream of N_2_ gas at 37°C, and the residue was re-dissolved in 100 μL of methanol and analyzed by LC-MS/MS. Vitamin D metabolites were separated on an Agilent 1100 series HPLC (Agilent Technologies, USA) and a C18 column. The isocratic mobile phase composition was A: 2 mmol/L ammonium acetate in H_2_O + 0.1% formic acid, B: 2 mmol/L ammonium acetate in methanol + 0.1% formic acid (10:90). The API4000 QTRAP mass spectrometer (Applied Biosystems/Sciex, Concord, ON, Canada) was equipped with a TurboV electrospray ion source operated in the positive mode. The ion transition of m/z 401.4→159.2 was monitored for 25-hydroxyvitamin D_3_, m/z 407.3→158.9 for D_6_-25-hydroxyvitamin D_3_ and m/z 413.4→107.2 for 25-hydroxyvitamin D_2_; however, none of the samples were found to contain detectable 25(OH)D2. This assay met the performance targets of the international Vitamin D External Quality Assessment Scheme (DEQAS) [21]. In a quality control subsample, between-run correlation was 0.95 and the mean coefficient of variation (CV%) was 9.9%. Data were reported using 30 nmol/L and 50 nmol/L cut-offs used in the IOM report [7] and the 80 nmol/L threshold used in the Hollis trial [13].

Serum/urine aliquots were maintained at 4°C and analyzed within 48 hours of collection for serum calcium, serum albumin, and urine calcium:creatinine ratio (ca:cr) on the AU640 Olympus Autoanalyzer (Olympus Corporation, Japan) in the Clinical Biochemistry Laboratory at icddr,b in Dhaka. Total serum calcium (Ca) concentration was adjusted for the serum albumin concentration by the following conventional formula: Ca + (0.02*(40-albumin)). Intact parathyroid hormone (PTH) was measured at baseline and delivery using a chemiluminescent microparticle immune assay (i1000SR Architect Autoanalyzer, Abbott, USA) at icddr,b.

### Safety monitoring

Reported severe symptoms, persistence of any mild symptomatic complaints for two consecutive visits, abnormal urinalyses, hypertension, or any other suspected pregnancy complications prompted referral by study personnel to the study physician for further evaluation. Biochemical data were reviewed daily by the study physician and at least weekly by the principal investigator. The upper limit of the serum calcium reference range used for safety monitoring was 2.60 mmol/L, which was conservative relative to the threshold for defining hypercalcemia used by the IOM in setting the 1997 DRIs for vitamin D (2.75 mmol/L) [10], and the threshold cited by the IOM committee in its 2010 revision of the vitamin D DRIs (2.63 mmol/L) [7]. Albumin-adjusted serum calcium concentration (adj-Ca) above 2.60 mmol/L prompted repeat blood sampling within 24 hours of the first sample report to the physician. ‘Confirmed hypercalcemia’, the primary supplement-related adverse event which would have prompted cessation of supplementation, was *a priori* defined as adj-Ca >2.60 mmol/L on two separate consecutive blood specimens. This definition was used because isolated values slightly above the upper limit of the reference range were not considered to be consistent with vitamin D toxicity. Urine ca:cr was expressed as mmol Ca/mmol Cr, considering 1.0 as the upper limit of the reference range [22]. Urine ca:cr was used as a screening test for possible hypercalcemia, such that unscheduled blood sampling to measure serum calcium concentration was performed if: two consecutive ca:cr > 1.0 mmol/mmol values; or, ca:cr > 1.0 mmol/mmol on two non-consecutive measurements but in the presence of persistent symptoms suggestive of possible hypercalcemia; or, ca:cr > 0.85 mmol/mmol on two consecutive urine specimens that was also ≥2-fold greater than the baseline value in the same participant. Participants with screen-positive urine ca:cr as defined by one of the above three criteria were also referred for renal ultrasound to rule-out the presence of nephrocalcinosis or urinary tract calculi. Participants were referred to an antenatal care physician at the maternity clinic for treatment of urinary tract infections, hypertension, or other medical problems diagnosed incidentally during follow-up. Participants with obstetric or neonatal complications were transported to a local tertiary-care hospital. Major clinical decisions (e.g., labour induction, cesarean deliveries) were the responsibility of non-study physicians at Shimantik or referral hospitals; however, costs of medical and obstetric care were borne by the study. A data and safety monitoring board (DSMB) met at regular intervals, but there were no *a priori* early stopping criteria (see Additional file 1 for a note regarding an interim DSMB review).

### Statistical analysis

The primary pharmacokinetic outcome was the serum 25(OH)D concentration and the primary safety measure was adj-Ca concentration. The primary analysis employed an intention-to-treat approach, including all data irrespective of a participant’s adherence or duration of supplementation. Data were visualized using cross-sectional density plots and longitudinal scatterplots with locally weighted regression (lowess), and described by means, standard deviations (SD) and 95% confidence intervals (95% CI), or median and interquartile range (IQR) for variables with skewed distributions. Analyses at 'delivery' were based on specimens collected within +/− 1 day of delivery. Urinary ca:cr at two weeks after enrollment was based on specimens collected 14 +/−1 days after the first supplement dose. Between-group differences in the mean maternal and cord serum 25(OH)D, maternal Ca, adj-Ca, and PTH, and maternal urinary ca:cr were analyzed at baseline and delivery using linear regression for cross-sectional comparisons and generalized estimating equations (GEE) for between-group differences in changes from baseline to delivery (to account for within-person correlation of paired measures). PTH and urinary ca:cr were log and square-root transformed, respectively, to approximate normality for parametric tests. Bootstrapping was employed to confirm the robustness of inferences. In per-protocol sensitivity analyses, we only included participants who received at least 8 consecutive weekly supplement doses and for whom delivery serum specimens were available.

Change in mean 25(OH)D over time was modeled as a continuous non-linear parametric function using a negative exponential growth function [16]. This approach enabled the estimation of the average difference in 25(OH)D between the two groups at a presumed ‘steady-state’ (i.e., where group-averaged 25(OH)D did not continue to appreciably rise despite ongoing vitamin D supplementation). As a per-protocol sensitivity analysis, change in mean 25(OH)D over time was modeled as a function of cumulative dose (micrograms) of vitamin D actually administered. Changes in serum Ca, adj-Ca, ca:cr, and PTH concentrations were modeled longitudinally using GEE with exchangeable correlation and robust variance estimation. Serum Ca, adj-Ca and urine ca:cr demonstrated non-linear trends over time in visual analysis by lowess plots; therefore, piecewise (spline) linear regressions were employed, with knot placement (the point on the x-axis where the piecewise regression functions join) for ca:cr ratio at day 14 (timing of scheduled urine collection) and at day 60 for serum Ca and adj-Ca. Knot placement was based on visual assessment of the trend indicated by the lowess functions. In sensitivity analyses, variation in knot placement did not change the inferences of the model coefficients (data not shown). Clinical outcomes were compared between groups using Student’s *t*-test or Chi-square and Fischer’s exact test. Frequencies of reported symptoms or minor clinical adverse events, based on weekly clinical monitoring, were analyzed using GEE where appropriate to estimate between-group differences. Given the large number of individual symptoms, the Holm procedure was used to correct for multiplicity of pair-wise comparisons [23]. Data were entered in an electronic database (SQL Server 2005) using a custom-designed interface (Visual Basic 6). Built-in range and consistency checks were employed in the data capture system to prevent errors in data entry. Analyses were conducted using Stata version 11 (Stata Corporation, College Station, Texas) and R version 2.15.1 (R Foundation for Statistical Computing, Vienna, Austria).

The target sample size was 160 pregnant women, to enable detection of a 0.5 SD difference in the primary outcome (serum 25(OH)D concentration at delivery), with a risk of a type I error rate (alpha) of 0.05, 80% power, and accounting for up to 20% attrition prior to delivery.

## Results

**Figure 1:**
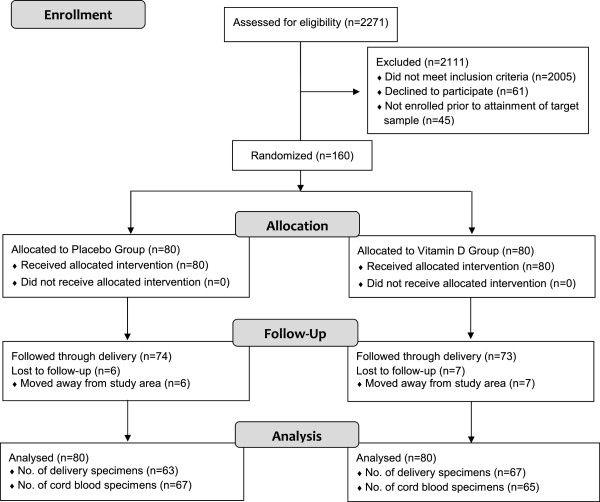
**CONSORT diagram depicting the flow of participants through the study.** Data for all subjects were analyzed at baseline. In longitudinal analyses, 74 participants in the placebo group and 77 participants in the vitamin D group contributed at least one biochemical value beyond baseline.

Of the 160 participants recruited and randomly assigned to either vitamin D (35,000 IU/week) or placebo, 13 were lost to follow-up prior to delivery (6 in the placebo group and 7 in the Vitamin D group), all because of having left the Dhaka area (Figure 1). Serum specimens were available for all participants at baseline and for 130 (81.3%) participants (63 placebo, 67 vitamin D) at delivery. Cord blood specimens were collected from 67 and 65 mother-infant pairs in the placebo and vitamin D groups, respectively. Maternal characteristics and 25(OH)D at baseline were similar in the placebo and vitamin D groups (Tables 1 and 2). Weekly supplementation occurred over a median period of approximately 10 weeks in both groups, and adherence was excellent among participants who contributed biochemical outcome data (Table 3).

**Table 1:** Participant baseline characteristics, overall and by treatment group

**Characteristic**	**Overall**	**Placebo**	**Vitamin D**
	**(n = 160)**	**(n = 80)**	**(n = 80)**
**Age, yrs**^**1**^	22.4 ± 3.5	22.4 ± 3.4	22.4 ± 3.5
**Gestational age, weeks**	27.8 ± 1.1	27.9 ± 1.0	27.6 ± 1.1
**Marital status**^**2**^			
Married	160 (100)	80 (100)	80 (100)
**Level of education**			
Primary school incomplete (<8 yrs)	102 (63.8)	50 (62.5)	52 (65.0)
High school incomplete (> = 8 to <12 yrs)	49 (30.6)	27 (33.8)	22 (27.5)
Graduate school (> = 12 yrs)	9 (5.7)	3 (3.8)	6 (7.5)
**Primary occupation**			
Homemaker	141 (88.1)	72 (90.0)	69 (86.3)
Other	19 (11.9)	8 (10.2)	11 (14.0)
**Number of pregnancies**			
Median	1	1	1
Range (Min, Max)	(1, 5)	(1, 4)	(1, 5)
**Number of live births**			
Median	0	0	0
Range (Min, Max)	(0, 4)	(0, 2)	(0, 4)
**Height, cm**	150.3 ± 5.1	150.4 ± 5.4	150.3 ± 4.9
**Weight, kg**	52.0 ± 8.5	51.8 ± 9.1	52.2 ± 8.0
**Body mass index (BMI)**	23.0 ± 3.3	22.8 ± 3.5	22.1 ± 3.1

^1^ Mean ± standard deviation (SD).

^2^ n (%).

**Table 2:** Serum 25(OH)D categories at baseline and delivery, by supplementation group

	**Placebo**^**1**^	**Vitamin D**^**1**^	***p***^**2**^
**Maternal Baseline**	**(n = 80)**	**(n = 80)**	
<30 nmol/L	21 (26.3)	18 (22.5)	0.88
30 – 49 nmol/L	32 (40.0)	32 (40.0)	
50 – 79 nmol/L	21 (26.3)	25 (31.3)	
> = 80 nmol/L	6 (7.5)	5 (6.3)	
**Maternal Delivery**	**(n = 63)**	**(n = 67)**	
<30 nmol/L	23 (35.9)	0 (0)	<0.001
30 – 49 nmol/L	27 (42.2)	0 (0)	
50 – 79 nmol/L	10 (15.9)	2 (3.0)	
> = 80 nmol/L	3 (4.8)	65 (97.0)	
**Cord blood**	**(n = 67)**	**(n = 65)**	
<30 nmol/L	21 (31.3)	1 (1.5)	<0.001
30 – 49 nmol/L	33 (49.3)	2 (3.1)	
50 – 79 nmol/L	9 (13.4)	7 (10.8)	
> = 80 nmol/L	4 (6.0)	55 (84.6)	

^1^ n (%).

^2^ p-value for Chi-square test or Fischer’s exact test for proportions.

**Table 3:** Supplementation duration and adherence

**Adherence**	**Placebo**			**Vitamin D**			***p***
	**Mean ± SD**	**Median**	**Range (min, max)**	**Mean ± SD**	**Median**	**Range (min, max)**	
**Time on study, weeks**	9.7 ± 3.5	10.5	(0, 18)	9.8 ± 3.3	10	(0, 18)	0.87^1^
**Total supplement doses administered**	10.5 ± 3.5	11	(1, 19)	10.6 ± 3.3	11	(1, 17)	0.83
**Total vitamin D administered, mcg**	-	-	-	9469 ± 2424	9625	(875, 14000)	-
**Adherence, %**^**2**^	99.2 ± 2.7	100	(84.6, 100)	99.4 ± 2.9	100	(80, 100)	0.79
**Participants who received 100% of scheduled doses, n (%)**^**3**^		68 (91.9%)			69 (94.5%)		0.53^3^

^1^ p-value for Student’s *t*-test for equality of means.

^2^ Adherence = (number of doses received divided by the number of doses scheduled) x 100. Analysis shown included participants who contributed 25(OH)D measurements beyond baseline (n = 74 and n = 73 in placebo and vitamin D groups, respectively). Adherence for participants for whom delivery specimens were available was similar: mean of 99.1% in the placebo group (n = 63) and 99.3% in the vitamin D group (n = 67). With respect to longitudinal analyses of changes in 25(OH)D over time, 206/219 serum specimens (93.5%) in the vitamin D group and 201/213 (94.4%) specimens in the placebo group were preceded by 100% dose adherence.

^3^ Proportions shown were calculated among participants who contributed 25(OH)D measurements beyond baseline (74 in placebo group or 73 in the vitamin D group); p value for chi-square test for proportions. Proportions in the subgroup of participants for whom delivery specimens were available were similar: 90.5% in the placebo group (n = 63) and 94.0% in the vitamin D group (n = 67).

**Figure 2:**
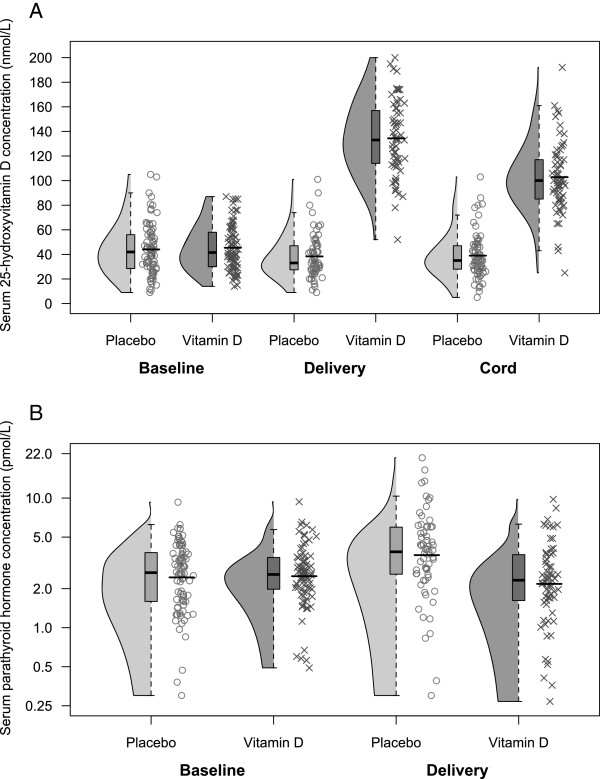
**Maternal and cord serum 25-hydroxyvitamin D and serum parathyroid hormone concentration at baseline and delivery.** (**A**) Maternal and cord serum 25-hydroxyvitamin D (25(OH)D) concentrations, and (**B**) maternal serum parathyroid hormone (PTH) concentrations. Kernel density plots, boxplots and jittered scatterplots illustrate the distribution of each subgroup. The limits of the box indicate the 25^th^ and 75^th^percentiles, and the black horizontal line within the box represents the median. (**A**) Black horizontal line within the scatterplot represents the mean; (**B**) the black horizontal line within the scatterplot represents the geometric mean.

**Figure 3:**
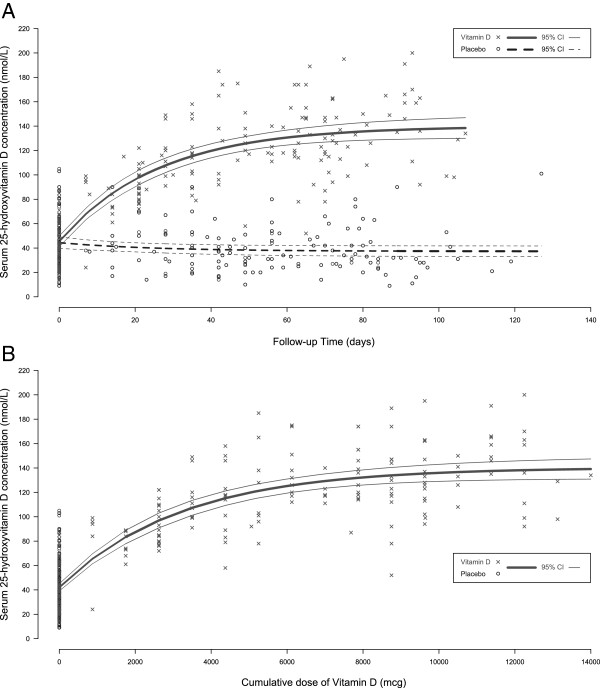
**Maternal serum 25-hydroxyvitamin D (25(OH)D) concentration, by time or cumulative dose of vitamin D3.** Thick lines represent the negative exponential model-predicted serum 25(OH)D concentrations as a function of time (**A**) and as a function of cumulative dose of vitamin D3 (**B**). Thin solid lines represent the 95% confidence interval (95% CI) around the predicted 25(OH)D. In panel **B**, all placebo group data are clustered at 0 mcg given the lack of vitamin D3 supplementation. Model fit as assessed by R^2^ was 0.75 in both panels **A** and **B**.

Most participants in both groups were vitamin D insufficient by the IOM threshold (25(OH)D <50 nmol/L) at baseline (Table 2). Vitamin D status at baseline did not differ between the supplementation groups (Table 4) nor between participants with and without delivery specimens (44.5 vs. 44.8 nmol/L, p = 0.94). Vitamin D supplementation had a substantial, statistically significant effect on maternal and neonatal vitamin D status at the time of delivery, as reflected in the overall 25(OH)D distributions (Figure 2; Table 4) as well as the proportion of participants considered to have low 25(OH)D using cut-offs (Table 2). Notably, 100% of mothers (at delivery) and 95% of neonates attained 25(OH)D >50 nmol/L compared to only 21% and 19% of mother and neonates, respectively, in the placebo group (Table 2). The highest 25(OH)D value in any participant was 200 nmol/L, observed in a vitamin D group participant at delivery; concurrently, her other biochemistry were normal (adj-Ca, 2.47 mmol/L; urine ca:cr, 0.23 mmol/mmol). Longitudinal analysis of the change in mean 25(OH)D over time revealed an early and rapid rise in 25(OH)D, with a steady-state approached after about 2 months of supplementation (Figure 3; Table 5). As evidence that this plateau was not an artifact of diminishing supplement adherence over time, a sensitivity analysis in which 25(OH)D was modeled as a function of cumulative vitamin D dose revealed the same pattern (Figure 3), and fit the data well (Table 5). Vitamin D status declined in the placebo group over time (Figure 3), but was not significantly lower at delivery compared to baseline (38.3 vs. 44.0, p = 0.09).

**Table 4:** Biochemical measures for maternal baseline, maternal delivery, and cord blood specimens, by supplementation group

**Biomarker (units)**	**Placebo**	**Vitamin D**	**Group difference **^**1**^		**Group-by-time effect**^**2**^	
	**(n = 80)**	**(n = 80)**	**Mean**	**95% CI**	**Mean**	**95% CI**
**25(OH)D (nmol/L)**						
Baseline^3^	44.0 ± 20.9	45.4 ± 18.4	1.4	[−4.8, 7.5]	–	–
Delivery (n = 130)^4^	38.4 ± 18.1	134.4 ± 30.7	96.0^***^	[87.6, 104.8]	94.6^***^	[85.0, 104.1]
Cord (n = 132)^5^	39.0 ± 18.7	102.8 ± 28.6	63.9^***^	[55.8, 72.0]	–	–
**Calcium (mmol/L)**						
Baseline	2.27 ± 0.09	2.25 ± 0.09	−0.02	[−0.04, 0.01]	–	–
Delivery (n = 130)	2.31 ± 0.11	2.32 ± 0.10	0.02	[−0.02, 0.05]	0.03	[−0.01, 0.07]
**Albumin-adjusted calcium (mmol/L)**						
Baseline	2.36 ± 0.07	2.35 ± 0.07	−0.01	[−0.03, 0.01]	–	–
Delivery (n = 130)	2.40 ± 0.08	2.43 ± 0.09	0.03^*^	[0.00, 0.06]	0.04^*^	[0.01, 0.07]
**PTH (pmol/L)**^**6**^						
Baseline	2.7 (0.3, 9.3)	2.9 (0.5, 9.4)	0.02	[−0.7, -0.2]	–	–
Delivery (n = 129)^7^	3.9 (0.3, 20.5)	2.3 (0.3, 9.8)	−0.51^***^	[−0.8, -0.3]	−0.53^***^	[−0.8, -0.3]
**Urinary calcium-creatinine ratio (mmol/mmol) **^**8**^						
Baseline	0.24 (0.0, 0.68)	0.22 (0.0, 0.88)	−0.01	[−0.07, 0.05]	–	–
2 weeks (n = 151)^9^	0.26 (0.0, 1.11)	0.26 (0.0, 1.25)	0.03	[−0.04, 0.10]	–	–
Delivery (n = 125)^10^	0.13 (0.0, 1.26)	0.20 (0.0, 2.26)	0.04	[−0.05, 0.14]	0.07	[−0.03, 0.16]

^1^ Mean difference [95% CI] between the placebo and vitamin D group at a given time, by ordinary least square estimation.

^2^ Mean difference [95% CI] in the change for vitamin D versus placebo group (difference in the slope) using generalized estimation equations (GEE) to account for within-subject correlations.

^3^ Mean ± standard deviation (SD).

^4^ Delivery specimens were those collected within +/−1 day of delivery; n = 63 and n = 67 in the placebo and vitamin D groups, respectively;

^5^ Cord serum available for n = 67 in the placebo and n = 65 in the vitamin D group.

^6^ Median (range) summarize maternal PTH concentrations, due to right-skewed distributions. PTH concentrations were log-transformed to approximate normality for regression analyses. The results presented as group differences are log-transformed PTH concentrations.

^7^ PTH concentration at delivery available for n = 63 in placebo group and n = 66 in the vitamin D group.

^8^ Median (Range) summarize Ca: Cr ratios in the first two columns. Ca: Cr ratios were square root-transformed to approximate normality for regression analyses; thus, the regression coefficients and confidence bounds are presented on a square-root scale.

^9^ Two-week specimens were defined as those collected 14 +/−1 days after enrolment.

^10^ Delivery urine samples were taken day of delivery, +/−1 day, n = 60 and n = 65 in the placebo and vitamin D groups, respectively.

*p-value < 0.05; **p-value 0.01; ***p-value < 0.001.

**Table 5:** Estimates of the change in serum 25(OH)D concentration over time and by cumulative vitamin D3 dose

**Model parameters (units)**	**[25(OH)D] over time (days) in Vitamin D group**^**1**^		**[25(OH)D] over time (days) among all participants**^**2**^		**[25(OH]D] by dose (mcg) among all participants**^**2**^	
	**β**	**95% CI [LB, UB]**	**β**	**95% CI [LB, UB]**	**β**	**95% CI [LB, UB]**
**Reference**	Day 0 (baseline)		Placebo group at Day 0 (baseline)		0 mcg of Vitamin D	
**25(OH)D at ref. point (nmol/L)**	45.5	[41.0, 49.9]	44.4	[39.7, 49.1]	42.1	[39.1, 45.1]
**Difference in 25(OH)D between groups at day 0 (nmol/L)**	–		1.1	[−5.3, 7.6]	–	
**Decay rate (days**^**-1 **^**or mcg**^**-1**^**)**	0.04	[0.03, 0.05]	0.04	[0.03, 0.05]	0.0003	[0.0002, 0.0004]
**Δ25(OH)D at steady-state in placebo group (nmol/L)**	–		−7.1	[−12.3, -1.9]	–	
**Effect of vitamin D on Δ25(OH)D at steady-state (nmol/L)**	94.4	[83.9, 104.9]	101.6	[89.7, 113.4]^3^	98.3	[88.7, 107.9]
**Attained steady-state 25(OH)D (nmol/L)**	139.9	[130.1, 149.6]	138.8	[127.4, 150.2]	140.4	[131.2, 149.6]
**Adjusted R**^**2**^	0.72		0.75		0.75	
**AIC**	–		3907.2		3904.4	

^**1**^80 participants in the vitamin D group, contributing 220 specimens. Negative exponential model (vitamin D group): [25(*OH*)*D*]_*t*_ = [25(*OH*)*D*]_*t* = 0_ + *a*_1_(1 − *e*^− *kt*^); where *t* is time, *a* is the Δ25(OH)D (where Δ is the change from baseline) at steady-state in the placebo group, and *k* is the decay rate to the asymptote.

^2^ The models include 160 participants overall (n = 80 in each group) contributing 433 specimens. Negative exponential model (both groups):

[25(*OH*)*D*]_*t*_ = [25(*OH*)*D*]_*t* = 0_ + *β* g + (*gd* + *a*_0_)(1 − *e*^− *kt*^); where *t* is time, *a* is the Δ25(OH)D at steady-state in the placebo group, *k* is the decay rate to asymptote, *g* is group assignment and *d* is the difference in Δ25(OH)D between groups at steady-state,

^**3**^ Difference in Δ25(OH)D between placebo and vitamin D groups at modeled steady-state.

Given the substantial inter-individual variability in the response to vitamin D supplementation (Figure 3), we explored the role of the following potential modifiers of the magnitude of the change in 25(OH)D (Δ25(OH)D) from baseline to delivery (selected *a priori*): maternal weight at baseline, maternal age, gestational age, maternal body mass index at baseline (BMI), gravidity, parity, baseline PTH status, season and baseline vitamin D status. None of these factors explained significant inter-individual variability in the change in 25(OH)D status (data not shown). Although Δ25(OH)D was inversely associated with baseline vitamin D status, and about 24% of the variance in Δ25(OH)D in the vitamin D group was explained by baseline 25(OH)D, this association appeared to be due to regression to the mean, since similar trends were observed in both the vitamin D and placebo groups. As expected, we found a strong correlation between maternal delivery and cord 25(OH)D (Pearson correlation = 0.87, p < 0.001), yet there was substantial variability among mother-infant pairs with respect to the cord-to-maternal ratio (ranged from 0.24 to 3.93). Notably, variation in cord 25(OH)D within the vitamin D group was not associated with duration of supplementation (p = 0.86) or cumulative vitamin D supplement dose administered (p = 0.93), indicating that the maternal-fetal vitamin D equilibrium was achieved rapidly.

**Figure 4:**
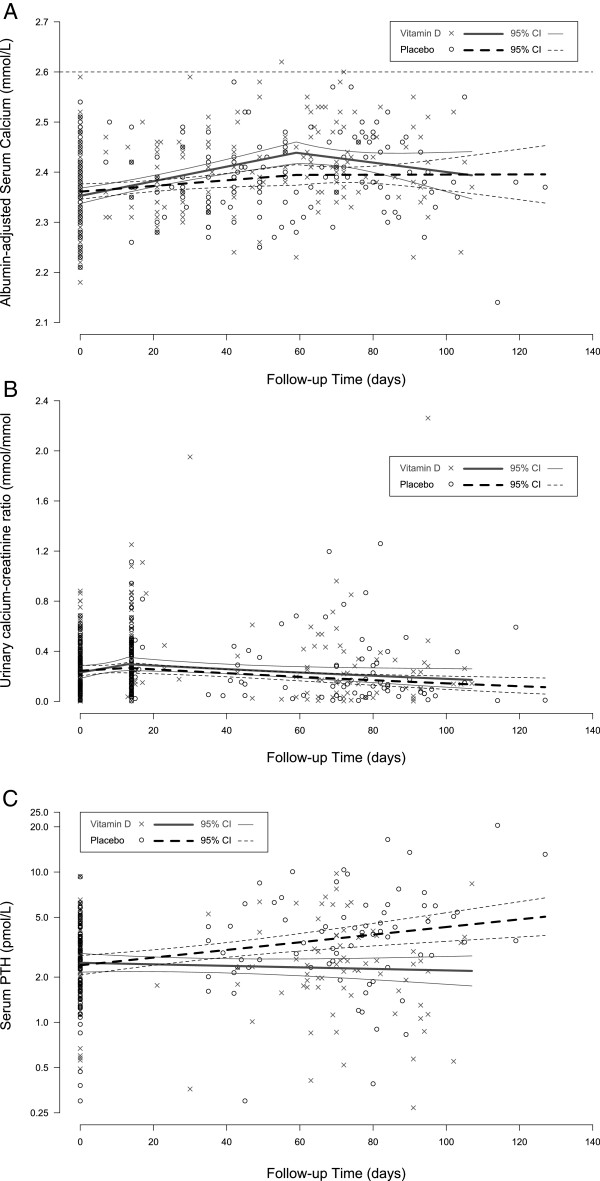
**Maternal albumin-adjusted serum calcium concentration (A), calcium:creatinine ratio (B), and serum parathyroid hormone concentration (C), by time.** Group means were modeled using piecewise linear regression (panels **A** and** B**) and generalized estimative equations (GEE). (**A**) Knot was placed at day 60, and the change in slope at that point was statistically significant in the vitamin D group (p < 0.01), but not in the placebo group. Associations: before knot in vitamin D group = 0.044 mmol/L per month, (p < 0.001); after knot in vitamin D group = −0.028 mmol/L per month, (p = 0.141); before knot in the placebo group = 0.017 mmol/L per month, (p < 0.01); after knot in the placebo group = 0.000 mmol/L per month, (p = 0.997). (**B**) Calcium:creatinine ratio was square-root transformed prior to regression analysis. A knot was placed at day 14, where the change in slope was statistically significant in the vitamin D group (p < 0.01), but not in the placebo group. Associations: before knot in the vitamin D group = 0.147 mmol/mmol per month, (p < 0.05); after knot in vitamin D group = −0.042 mmol/mmol per month, (p < 0.05); before knot in placebo group = 0.052 mmol/mmol per month, (p = 0.311); after knot in placebo group = −0.048 mmol/mmol per month, (p < 0.01). Varying the knot location in sensitivity analyses did not substantially change the inferences from panels **A** or **B**. (**C**) Parathyroid hormone concentrations were log transformed prior to regression analyses. There was a significant increase over time in the placebo group (0.01 log-pmol/L [95% CI: 0.003, 0.008] for each day increase in follow-up time; p < 0.001), but the slope of the vitamin D group was significantly attenuated (0.007 log-pmol/L [95% CI: -0.01, -0.004] lower than in the placebo group; p < 0.001).

**Figure 5:**
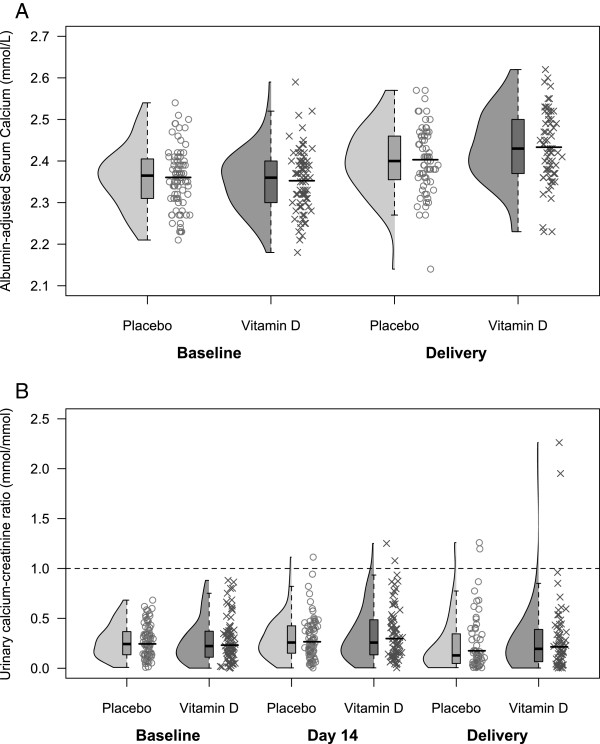
**Maternal albumin-adjusted serum calcium (A) and urine calcium:creatinine ratio (B), at baseline, day 14 and delivery.** Specimens were collected at baseline, 14 days after enrolment (for urine ca:cr) and at delivery. Kernel density plots, boxplots and jittered scatterplots illustrate the distribution of each subgroup. Upper and lower bounds of the box illustrate the 25^th^ and 75^th^ percentiles, and the black horizontal line within the box represents the median. (**A**) The black horizontal line within the scatterplot represents the mean; (**B**) black horizontal line within the scatterplot represents the square of the mean of square root-transformed ca:cr.

Mean maternal adj-Ca concentration rose over time within the reference range; the initial rate of rise appeared to be greater in the vitamin D group, but the difference was attenuated after ~2 months of supplementation (Figure 4). The mean adj-Ca at delivery and the change in this parameter from baseline to delivery were slightly but significantly greater in the vitamin D group versus placebo (Table 4; Figure 5). Conversely, the between-group differences in the mean total unadjusted serum Ca concentration at delivery and the change from baseline to delivery were of lower magnitude and not significant (Table 4). There were no cases of hypercalcemia in either group, defined *a priori* as adj-Ca >2.60 nmol/L on two separate consecutive blood specimens. One participant in the vitamin D group exhibited an isolated adj-Ca value of 2.62 mmol/L at the time of delivery, in the context of an acute diarrheal illness. Repeat measurements were 2.45 mmol/L on both days 3 and 12 postpartum (see Additional file 2: Table S3 for biochemical adverse events at any time during follow-up); therefore, trial criteria for hypercalcemia were not met. This participant’s unadjusted total serum Ca measurements were 2.48 mmol/L at delivery, 2.19 mmo/L at 3 days postpartum, and 2.41 mmol/L at 12 days postpartum. Her calcium:creatinine ratio ranged from 0.02 to 0.22 mmol/mmol and 25(OH)D ranged from 22 to 118 nmol/L. In secondary post-hoc analyses using all maternal serum specimens in which 25(OH)D and calcium were measured simultaneously, adj-Ca was significantly associated with 25(OH)D at 25(OH)D < 100 nmol/L (0.02 mmol/L [95% CI: 0.01, 0.03] increase in adj-Ca per 25 nmol/L increase in 25(OH)D below 100 nmol/L; n = 331), but this association was nullified and non-significant at 25(OH)D > =100 nmol/L (−0.01 mmol/L [95% CI: -0.02, 0.01] per 25 nmol/L increase in 25(OH)D above 100 nmol/L; n = 102).

Urinary ca:cr rose marginally in both groups after the first two weeks of supplementation, but declined thereafter (Figure 4). Changes over time were similar in both groups (Figure 4), and there were no significant differences in ca:cr between the two groups at delivery (Table 4; Figure 5). Hypercalciuria, defined *a priori* as ca:cr >1.0 mmol/mmol on 2 consecutive separate occasions, occurred in one participant in the vitamin D group, at 29 weeks gestation on day 14 of follow-up. Ca:cr was 1.08 mmol/mmol on day 14 and 1.11 mmol/mmol on day 16, but was normal at delivery (0.23 mmol/mmol) after continuation of vitamin D supplementation for a total of 10 weeks (cumulative dose of 9625 mcg). Throughout follow-up, this participant’s adj-Ca was within the reference range (2.32 to 2.55 mmol/L) and 25(OH)D concentration ranged from 54 – 147 nmol/L. A renal ultrasound showed an absence of nephrocalcinosis or renal calculi, and there were no associated clinical consequences. Transient hypercalciuria, defined as one ca:cr measurement above 1.0 mmol/mmol, was observed in 3 (4.1%) participants in the placebo group and 4 (5.5%) in the vitamin D group (*p* = 0.72 for between-group difference). Similarly, the proportion of participants with a ca:cr >0.8 mmol/mmol and >2-fold difference from baseline did not differ significantly between the two groups (2.7% vs. 5.5%, *p* = 0.44).

Vitamin D supplementation had a significant suppressive effect on maternal PTH concentration, such that the rise in PTH observed in the placebo group was completely attenuated in the vitamin D group (Figure 4), and the average PTH concentration was lower in the vitamin D group at delivery (Figure 2; Table 4). Detailed weekly clinical monitoring did not reveal significant differences in reported symptom or minor adverse event frequencies between the two groups (online supplemental material). In particular, the overall proportion of encounters in which there was at least one symptom possibly suggestive of hypercalcemia was similar for the placebo and vitamin D groups (54% vs. 53%, *p* = 0.71; analysis accounted for repeated events within the same participants). Major pregnancy/birth outcome and serious clinical adverse event frequencies and distributions were similar across the two groups (Table 6).

**Table 6:** Pregnancy outcomes and neonatal anthropometry, overall and by study group

**Outcome**	**Overall**	**Placebo**	**Vitamin D**	***p***^**2**^
	**(n = 147)**	**(n = 74)**	**(n = 73)**	
**Location of delivery**^**1**^				
Shimantik Maternity centre	124 (84.4)	62 (83.8)	62 (84.9)	1.00
Home	13 (8.8)	7 (9.7)	6 (8.2)	
Other hospital or clinic	1 (0.7)	0 (0)	1 (1.4)	
Other	9 (6.1)	5 (6.8)	4 (5.5)	
**Mode of delivery**				
Vaginal	59 (40.1)	30 (40.5)	29 (39.7)	1.00
C-section	88 (59.7)	44 (59.7)	44 (60.3)	
**Vaginal delivery**^**3**^				
Spontaneous	54 (91.5)	28 (93.3)	26 (89.7)	0.80
Forceps	3 (5.1)	1 (3.3)	2 (6.9)	
Unknown	2 (3.4)	1 (3.3)	1 (3.5)	
**Indication for c-section**^**4**^				
Urgent	76 (81.7)	41 (89.1)	35 (74.5)	0.17
Elective	11 (11.8)	3 (6.5)	8 (17.0)	
Unknown	6 (6.5)	2 (4.4)	4 (8.5)	
**Maternal serious clinical adverse events**^**5**^	4 (2.5)	2 (2.7)	2 (2.7)	1.00
**Gestational age at birth, wks**^**6**^	38.4 ± 2.1	38.5 ± 2.1	38.2 ± 2.1	0.31
**Infant anthropometry**				
Birth weight, g^7^	2795 ± 467	2788 ± 378	2802 ± 543	0.86
Length at birth, cm^8^	48.1 ± 2.5	48.0 ± 2.0	48.2 ± 2.5	0.55
Head circumference, cm^9^	33.0 ± 1.7	33.0 ± 1.5	32.9 ± 1.8	0.71
**Total births registered**^10^	147 (91.9)	74 (92.5)	73 (91.3)	0.77
Live births^11^	145 (98.6)	73 (98.6)	72 (98.6)	0.99
Stillbirths^11^	2 (1.4)	1 (1.4)	1 (1.4)	1.00
**Neonatal clinical adverse events**				
Neonatal serious non-fatal adverse events	13 (8.8)	7 (9.5)	6 (8.2)	0.81
Neonatal deaths	4 (2.8)	3 (4.1)	1 (1.4)	0.62

^1^ n (%).

^2^ p-value for Student’s *t*-test for means and Fischer’s exact test for proportions, conducted for variables with cell counts <5.

^3^ n = 59, as mode of delivery was vaginal.

^4^ n = 88, as mode of delivery was c-section.

^5^ Maternal serious adverse events included gastroenteritis, preterm delivery with premature rupture of membranes, injury (15% flame burn), and pre-eclampsia.

^6^ Mean ± standard deviation (SD).

^7^ Birth weight was missing for 2 infants in the placebo group and 1 in the vitamin D group.

^8^ n = 137 (69 in placebo, 68 in the vitamin D group).

^9^ n = 132 (65 in placebo, 67 in vitamin D group).

^10^ Denominator for calculation of proportions was the total number of women enrolled (n = 160).

^11^ Denominator for calculation of proportions was the total number of births registered (n = 147).

## Discussion

Among pregnant women with relatively low average baseline vitamin D status in Dhaka, 3rd-trimester vitamin D3 supplementation (35,000 IU/week) significantly raised maternal and neonatal (cord blood) serum 25(OH)D concentrations above the IOM cut-off for sufficiency (50 nmol/L) in virtually all participants without inducing hypercalcemia or other apparent short-term clinical adverse effects. This study contributes pharmacokinetic data relevant to South Asia, as well as preliminary evidence in support of a vitamin D3 no observed adverse effect level (NOAEL) of 35,000 IU/week in the third trimester of pregnancy.

The present findings build primarily on those of Hollis et al. in South Carolina, who randomized 502 pregnant women at 12 to 16 weeks gestation to receive vitamin D3 at doses of 400 IU/day, 2000 IU/day, or 4000 IU/day; 350 participants (70%) were followed-up until delivery [13]. From a baseline 25(OH)D of 58 nmol/L, 2000 IU/day and 4000 IU/day raised 25(OH)D to means at the time of delivery of 98 nmol/L (rise of 40 nmol/L) and 111 nmol/L (rise of 52 nmol/L), respectively [13]. In the 4000 IU/day group, 82% attained 80 nmol/L at delivery, compared to 97% in the present study (5000 IU/day). In 2012, Dawodu et al. presented unpublished findings from a trial in United Arab Emirates in which 2000 IU/day and 4000 IU/day raised mean 25(OH)D from ~20 nmol/L to ~65 nmol/L (rise of 45 nmol/L) and ~90 nmol/L (rise of 70 nmol/L) at delivery, respectively [24]. Our modeled mean increment in maternal 25(OH)D of 102 nmol/L corresponded to a rise of 0.82 nmol/L/mcg/day (95% CI, 0.72 to 0.91), which was similar to the value often cited for non-pregnant adults (~0.7 nmol/L/mcg/day [25,26]) but smaller than the effect observed with lower vitamin D3 doses (e.g., 1.6 nmol/L/mcg/day for doses of ~800 IU/day [27]). This is consistent with the hypothesized constraint in hepatic vitamin D–to–25(OH)D conversion that occurs at a 25(OH)D concentration of about 80–90 nmol/L [28].

In a preceding pilot trial in Dhaka, we found that 3^rd^-trimester regimens of 14,000 IU/week (≈2000 IU/day) and 35,000 IU/week (≈5000 IU/day) led to mean 25(OH)D concentrations of 76 nmol/L (rise of 36 nmol/L) and 98 nmol/L (rise of 57 nmol/L), respectively, after 10 weeks of supplementation [16]. Thus, compared to the present study, the average response to 35,000 IU/week was less pronounced in the pilot study. However, direct between-study comparisons are tempered by differences in study design (e.g., shorter period of follow-up in the pilot study) and 25(OH)D assays. In comparison to the LC-MS/MS method used here, the immunoassay used in the pilot study (Diasorin Liaison Total) may have under-estimated 25(OH)D because of raised DBP concentrations in pregnancy [29] or relative imprecision at the higher 25(OH)D range attained in the vitamin D group [30,31].

Similar to previous trials [13,16], we observed substantial inter-individual variability in the maternal Δ25(OH)D, such that the SD on the mean 25(OH)D nearly doubled from 18.4 nmol/L at baseline to 30.7 nmol/L at delivery in the vitamin D group; in contrast, in the placebo group, the SD at baseline was 20.1 nmol/L and at delivery was 18.1 nmol/L. However, we could not identify any participant factor other than intervention group that significantly explained Δ25(OH)D. The range of published mean cord blood 25(OH)D concentrations in South Asia, from 21 [32] to 59 [33] nmol/L, bounds the mean cord 25(OH)D of 39 nmol/L in the control group. A normal cord 25(OH)D range based on functional outcomes has not been defined; however, Zeghoud et al. (1997) reported that newborn 25(OH)D > 30 nmol/L was associated with suppression of PTH concentrations [34], and 25(OH)D > 50 nmol/L (the 25(OH)D threshold for sufficiency set by the IOM) has been associated with a low risk of rickets [35]. In our study, 95% of newborns had a cord-maternal 25(OH)D ratio of 0.5 to 1.5, suggesting that universal maternal prenatal 25(OH)D > 100 nmol/L would ensure that nearly all cord concentrations are above 50 nmol/L.

We remain guarded in our interpretation of these pharmacokinetic data because the clinical implications of the observed changes in maternal-fetal 25(OH)D and resultant maternal PTH suppression in pregnancy remain to be defined. Hollis et al. concluded from their trial findings “that the current vitamin D EAR and RDA for pregnant women issued in 2010 by the IOM should be raised to 4000 IU of vitamin D per day” [13]. However, we do not believe that above-RDA doses should yet be recommended as routine practice. Further clinical data are required before conclusive statements can be made regarding the appropriateness of high-dose antenatal vitamin D supplementation. However, like Hollis et al., we did not encounter any evidence of vitamin D toxicity. Vitamin D did not affect average urinary calcium excretion, yet we did see a small but significant increase in the mean adj-Ca in the vitamin D group compared to the placebo group. Several factors suggested this was not likely to be evidence of harm. First, this trend was attenuated and non-significant when the analysis involved uncorrected serum calcium, a less conservative endpoint used in previous trials. Second, we did not observe a linear escalation in adj-Ca over the entire duration of supplementation, but rather found an initial increase in the rate of the natural rise in adj-Ca that occurs in late pregnancy [36] followed by an attenuation of the between-group difference after about 2 months of supplementation. This suggested a homeostatic adjustment to the altered vitamin D status, with no further increase in serum calcium after a steady-state 25(OH)D was reached. Third, a positive association between adj-Ca and 25(OH)D was apparent in the range of 25(OH)D below 100 nmol/L but not above 100 nmol/L; evidence of possible harm would be more consistent with the opposite finding of a serum calcium that was tightly controlled (i.e., unassociated with 25(OH)D) within the lower range of 25(OH)D, but deflected upward at higher 25(OH)D. All maternal uncorrected and adj-Ca values were below the threshold for hypercalcemia used by the IOM in the 1997 vitamin D DRIs (2.75 mmol/L) [10] and the 2010 revision (2.63 mmol/L) [7]. We set a conservative upper limit of reference range for trial monitoring (2.60 mmol/L), which was crossed by one participant in the vitamin D group; however, this was not logically attributable to vitamin D toxicity based on normal repeat serum calcium, normal ca:cr, and a 25(OH)D that was well within the range considered to be non-hypercalcemic [37]. Based on biochemical findings, and the lack of apparent clinical adverse effects, we cautiously conclude that 35,000 IU/week for an average of 10 weeks (up to 18 weeks) was tolerated by study participants, and could be considered an upper dose limit for use in future research.

This RCT had several limitations. It was not designed to draw precise inferences regarding pregnancy/birth outcomes, which limits its clinical applications. The biochemical dose–response curve would have been improved by the inclusion of other dose levels. Because of the high dose of vitamin D not previously studied in pregnancy, enrolment was limited to healthy women at low risk of pregnancy complications, and who were most likely to adhere to the protocol; as such, generalizability of the findings may be limited. In addition, enrolment was not conducted throughout the entire season, but rather occurred during a period in which vitamin D status was declining from its summer peak. Mechanistic inferences were limited because we did not measure serum concentrations of 1,25(OH)2D, vitamin D3, or DBP. As well, we did not calculate total intake of vitamin D due to the lack of adequate information regarding vitamin D content in the Bangladeshi food supply.

## Conclusions

In conclusion, this RCT in Dhaka enabled a detailed characterization of the biochemical response to 35,000 IU/week vitamin D3 supplementation in the third-trimester of pregnancy. Future studies involving equal or lesser doses are required to establish maternal-infant benefits and longer-term safety.

## Abbreviations

AViDD: Antenatal Vitamin D in Dhaka; 25(OH)D: 25-Hydroxyvitamin D; IU: International Units; IOM: Institute of Medicine; DRI: Dietary Reference Intake; RDA: Recommended Dietary Allowance; UL: Tolerable Upper Intake Level; UNICEF: United Nations International Children’s Emergency Fund; RCT: Randomized Controlled Trial; icddr, b: International Centre for Diarrhoeal Disease Research, Bangladesh; LC-MS/MS: Liquid Chromatography Tandem Mass Spectroscopy; HPLC: High-Performance Liquid Chromatography; DEQAS: Vitamin D External Quality Assessment Scheme; CV: Coefficient of Variation; Ca:Cr: Calcium: Creatinine Ratio (mmol/mmol); Ca: Serum Calcium; PTH: Parathyroid Hormone; adj-Ca: Albumin-Adjusted Serum Calcium; DSMB: Data and Safety Monitoring Board; GEE: Generalized Estimating Equations; NOAEL: No Observed Adverse Effect Level; 1,25(OH)2D: 1,25-dihydroxyvitamin D; DBP: Vitamin D Binding Protein.

## Competing interests

The authors declare that they have no competing interests.

## Authors’ contributions

DR, AM, and AB designed the research; DR, AM, RR, and EA conducted the research; DR, NP and BP performed the data analyses. DR wrote the manuscript and had the primary responsibility for the final content. All authors read and approved the final manuscript.

## Supplementary Material

Additional file 1 **Note regarding an interim DSMB review.** This note describes an interim review conducted by the data and safety monitoring board (DSMB). Click here for file

Additional file 2 **AViDD trial sensitivity analyses, weekly symptoms reported, and biochemical and safety data.** This file includes summaries of intention-to-treat analyses versus sensitivity analyses (**Table S1**), frequency of reported symptoms based on weekly clinical monitoring (**Table S2**), biochemical adverse events (**Table S3**) and clinical serious adverse events (**Table S4**) at any time during follow-up, and biochemical and clinical data among newborns with clinical serious adverse events (birth to 1 month postnatal). Click here for file
