# Supplementary material for: Randomized placebo-controlled trial of high-dose prenatal third-trimester vitamin D3 supplementation in Bangladesh: the AViDD trial
Source: Nutr J. 2013 Apr 12;12:47. doi: 10.1186/1475-2891-12-47 (PMC3641012; doi:10.1186/1475-2891-12-47)
Supplement: Additional file 1 — Note regarding an interim DSMB review. This note describes an interim review conducted by the data and safety monitoring board (DSMB). [file 1475-2891-12-47-S1.pdf]

Note regarding an interim DSMB review

On December 14, 2010, an observational sub-study of neonatal calcium homeostasis was initiated among infants born to participants in the AViDD trial. Procedures implemented for the sub-study (e.g., blood sampling in the first week of life) were not intended to affect the primary trial aims or outcome assessment. At the time of initiation of the sub-study, 116 (of 160) pregnant participants had already been enrolled, and 50 pregnancies had already been completed.

On February 1, 2011, based on the observation that some infants enrolled in the sub-study had asymptomatic serum calcium concentrations above the pre-stated reference range, the investigators requested that the icddr,b ethical review committee (ERC) and data and safety monitoring board (DSMB) consider the implication of these findings for continuation of vitamin D supplementation among pregnant participants still under study. At that time, enrollment of all 160 participants was completed, and 94 pregnancies were completed.

On February 14, 2011, the ERC reported the unblinding of 6 affected infants. One infant's group assignment was revealed to the investigators, whereas allocation of the other 5 were reported in aggregate; overall, 3/6 affected infants were in the vitamin D group and 3/6 in the placebo group. All infants were clinically well.

The ERC and DSMB approved continuation of the trial. In a memo on March 21, 2011, the DSMB summarized their review of the events, and concluded that, "these events were of unclear significance and did not present immediate harm to the participants" and reiterated their approval of the continuation of the trial.

Further evaluation of these events by study investigators revealed an absence of evidence of vitamin D toxicity. Results of the neonatal calcium sub-study will be presented elsewhere.
