# Supplementary material for: Randomized placebo-controlled trial of high-dose prenatal third-trimester vitamin D3 supplementation in Bangladesh: the AViDD trial
Source: Nutr J. 2013 Apr 12;12:47. doi: 10.1186/1475-2891-12-47 (PMC3641012; doi:10.1186/1475-2891-12-47)
Supplement: Additional file 2 — AViDD trial sensitivity analyses, weekly symptoms reported, and biochemical and safety data. This file includes summaries of intention-to-treat analyses versus sensitivity analyses (Table S1), frequency of reported symptoms based on weekly clinical monitoring (Table S2), biochemical adverse events (Table S3) and clinical serious adverse events (Table S4) at any time during follow-up, and biochemical and clinical data among newborns with clinical serious adverse events (birth to 1 month postnatal). [file 1475-2891-12-47-S2.pdf]

Table S1: Biochemical measures for maternal delivery, and cord blood specimens, overall and by supplementation group, comparing ITT and protocol sensitivity analyses restricted to women who received ≥8 doses and not lost to follow-up.

Biomarker (units)	Placebo (n = 80)	Vitamin D (n = 80)	Group Difference [1]		Group-by-Time Effect [2]	
			Mean	95% CI	Mean	95% CI
**25(OH)D (nmol/L)**						
Delivery (n=130)[3]	38.4 ± 18.1	134.4 ± 30.7	96.0***	[87.6, 104.8]	94.6***	[85.0, 104.1]
Delivery (n=115)[4]	37.8 ± 19.0	134.8 ± 30.3	97.0***	[88.2, 105.9]	96.2***	[86.1, 106.4]
Cord (n=132)[5]	39.0 ± 18.7	102.8 ± 28.6	63.9***	[55.8, 72.0]	–	–
Cord (n=116)[6]	39.3 ± 19.7	100.8 ± 26.6	61.1***	[52.9, 69.3]	–	–
**Calcium (mmol/L)**						
Delivery (n=130)	2.31 ± 0.11	2.32 ± 0.10	0.02	[-0.02, 0.05]	0.03	[-0.01, 0.07]
Delivery (n=115)	2.31 ± 0.11	2.32 ± 0.10	0.01	[-0.03, 0.05]	0.03	[-0.01, 0.07]
**Albumin-adjusted calcium (mmol/L)**						
Delivery (n=130)	2.40 ± 0.08	2.43 ± 0.09	0.03*	[0.00, 0.06]	0.04*	[0.01, 0.07]
Delivery (n=115)	2.40 ± 0.08	2.43 ± 0.09	0.03	[0.00, 0.06]	0.04*	[0.01, 0.07]
**PTH (pmol/L)[7]**						
Delivery (n=129)[8]	3.9 (0.3, 20.5)	2.3 (0.3, 9.8)	-0.51***	[-0.8, -0.3]	-0.53***	[-0.8, -0.3]
Delivery (n=115)[4]	4.0 (0.4, 20.5)	2.4 (0.3, 9.8)	-0.54***	[-0.8, -0.3]	-0.58***	[-0.9, -0.3]
**Urinary calcium-creatinine ratio (mmol/mmol) [9]**						
Delivery (n=125)[10]	0.13 (0.0, 1.26)	0.20 (0.0, 2.26)	0.04	[-0.05, 0.14]	0.07	[-0.03, 0.16]
Delivery (n=110)[11]	0.12 (0.0, 1.26)	0.17 (0.0, 2.26)	0.03	[-0.07, 0.13]	0.06	[-0.04, 0.16]

[1] Mean difference [95% CI] between the placebo and vitamin D group at a given time, by ordinary least square estimation.
[2] Mean difference [95% CI] in the change for vitamin D versus placebo group (difference in the slope) using generalized estimation equations (GEE) to account for within-subject correlations.
[3] Mean ± standard deviation (SD); Delivery specimens were those collected within +/-1 day of delivery; n=63 and n=67 in the placebo and vitamin D groups, respectively;
[4] Delivery specimens collected +/- 1day of delivery after at least 8 cumulative doses of vitamin D, excluding women lost to follow up; n=54 and n=61 in the placebo and vitamin D groups.
[5] Cord serum available for n=67 in the placebo and n=65 in the vitamin D group.
[6] Cord serum available for n=56 and n=60 in the placebo and vitamin D groups, respectively, for women who received at least 8 cumulative doses.
[7] Median (range) summarize maternal PTH concentrations, due to right-skewed distributions. PTH concentrations were log-transformed to approximate normality for regression analyses. The results presented as group differences are log-transformed PTH concentrations.
[8] PTH concentration at delivery available for n=63 in placebo group and n=66 in the vitamin D group.
[9] Median (Range) summarize Ca:Cr ratios in the first two columns. Ca:Cr ratios were square root-transformed to approximate normality for regression analyses; thus, the regression coefficients and confidence bounds are presented on a square-root scale.
[10] Delivery urine samples were taken day of delivery, +/-1 day, n=60 and n=65 in the placebo and vitamin D groups, respectively.
[11] Per protocol analyses delivery urine samples were taken day of delivery, +/-1 day, n=51 and n=59 in the placebo and vitamin D groups, respectively.
*p-value <0.05; **p-value 0.01; ***p-value< 0.001

Table S2: Frequencies of reported symptoms based on weekly clinical monitoring

Symptoms	Total No. of Reported Events[1]			Reported at baseline visit[2]			Ever reported during follow-up[2]			Ever reported after 60 days of follow-up[2]		
	Placebo	Vitamin D	*p*[3]	Placebo	Vitamin D	*p*[4]	Placebo	Vitamin D	*p*[5]	Placebo	Vitamin D	*p*[6]
**Decreased appetite**	19 (2.3)	15 (1.8)	0.60	7 (8.8)	6 (7.5)	0.77	8 (10.0)	7 (8.8)	0.78	2 (2.5)	1 (1.3)	0.62
**Vomiting**	71 (8.6)	85 (10.2)	0.36	10 (12.5)	11 (13.8)	0.82	36 (45.0)	31 (38.8)	0.51	11 (13.8)	6 (7.5)	0.22
**Fever or chills**	26 (3.1)	15 (1.8)	0.10	2 (2.5)	2 (2.5)	1.0	17 (21.3)	11 (13.8)	0.24	4 (5.0)	2 (2.5)	0.41
**Constipation**	48 (5.8)	62 (7.4)	0.42	13 (16.3)	14 (17.5)	0.83	24 (30.0)	22 (27.5)	0.74	7 (8.75)	9 (11.3)	0.63
**Diarrhea**	20 (2.4)	5 (0.6)	0.01	3 (3.8)	1 (1.3)	0.62	13 (16.3)	4 (5.0)	0.03	3 (3.8)	1 (1.3)	0.37
**Abdominal pain**	114 (13.8)	149 (17.8)	0.20	4 (5.0)	6 (7.5)	0.75	49 (61.3)	49 (61.3)	0.96	25 (31.3)	26 (32.5)	0.91
**Cough**	81 (9.8)	118 (14.1)	0.08	7 (8.8)	12 (15.0)	0.22	35 (43.8)	42 (52.5)	0.44	10 (12.5)	17 (21.3)	0.18
**Difficulty breathing**	13 (1.6)	12 (1.4)	0.73	1 (1.25)	0	1.0	8 (10.0)	8 (10.0)	0.99	3 (3.8)	2 (2.5)	0.69
**Excessive thirst**	78 (9.4)	83 (9.9)	0.80	19 (23.8)	17 (21.3)	0.71	24 (30.0)	25 (31.3)	0.91	4 (5.0)	6 (7.5)	0.75
**Frequent urination**	200 (24.1)	184 (22.0)	0.61	38 (47.5)	31 (38.8)	0.26	47 (58.8)	43 (53.8)	0.63	16 (20.0)	14 (17.5)	0.69
**Burning sensation or pain during urination**	20 (2.4)	18 (2.2)	0.90	3 (3.8)	3 (3.8)	1.0	12 (15.0)	9 (11.3)	0.50	2 (2.5)	2 (2.5)	1.0
**Muscle weakness**	15 (1.8)	10 (1.2)	0.44	1 (1.3)	0	1.0	10 (12.5)	7 (8.8)	0.45	3 (3.8)	2 (2.5)	1.0
**Back pain or cramps**	167 (20.1)	173 (20.7)	0.99	11 (13.8)	8 (10.0)	0.46	50 (62.5)	48 (60.0)	0.80	27 (33.8)	26 (32.5)	0.86
**Leg pain or cramps**	78 (9.4)	85 (10.2)	0.77	5 (6.3)	2 (2.5)	0.25	29 (36.3)	35 (43.8)	0.47	14 (17.5)	11 (13.8)	0.53
**Arm pain or cramps**	18 (2.2)	8 (1.0)	0.10	1 (1.3)	0	1.0	11 (13.8)	7 (8.8)	0.33	3 (3.8)	0	0.12
**Mental confusion**	0	0	-	0	0	-	0	0	-	0	0	-
**Depressed mood**	0	1 (0.1)	-	0	0	-	0	1 (1.3)	1.0	0	0	-
**Severe headache**	25 (3.0)	23 (2.8)	0.81	3 (3.8)	1 (1.3)	0.62	12 (15.0)	14 (17.5)	0.71	4 (5.0)	6 (7.5)	0.75
**Blurry vision during the day**	4 (0.5)	11 (1.3)	0.30	0	2 (2.5)	0.50	4 (5.0)	4 (5.0)	1.0	2 (2.5)	0	0.25
**Difficulty seeing at night**	1 (0.1)	11 (1.3)	0.05	0	1 (1.3)	1.0	1 (1.3)	2 (2.5)	1.0	0	1 (1.3)	1.0
**Fainting**	4 (0.5)	0	-	0	0	-	3 (3.8)	0 (0)	0.08	0	0	-
**Convulsions**	0	0	-	0	0	-	0	0	-	0	0	-
**Vaginal bleeding**	1 (0.1)	1 (0.1)	-	0	0	-	1 (1.3)	1 (1.3)	1.0	1 (1.3)	0	0.50
**Malodorous or discoloured vaginal discharge**	4 (0.5)	5 (0.6)	0.73	0	0	-	4 (5.0)	4 (5.0)	1.0	3 (3.8)	1 (1.3)	0.37
**Clear vaginal fluid**	101 (12.2)	91 (10.9)	0.56	21 (26.3)	15 (18.8)	0.26	30 (37.5)	36 (45.0)	0.47	5 (6.3)	11 (13.8)	0.14
**Swelling of hands and feet**	29 (3.5)	17 (2.0)	0.31	0	1 (1.3)	1.0	15 (18.8)	8 (10.0)	0.14	7 (8.8)	3 (3.8)	0.22
**Bruising**	305 (36.8)	311 (37.2)	0.72	17 (21.3)	13 (16.3)	0.42	49 (61.3)	46 (57.5)	0.72	34 (42.5)	31 (38.8)	0.68

Symptoms	Total No. of Reported Events[1]			Reported at baseline visit[2]			Ever reported during follow-up[2]			Ever reported after 60 days of follow-up[2]		
	Placebo	Vitamin D	*p*[3]	Placebo	Vitamin D	*p*[4]	Placebo	Vitamin D	*p*[5]	Placebo	Vitamin D	*p*[6]
**Bleeding from rectum or in urine**	0	4 (0.5)	-	0	0	-	0	1 (1.3)	1.0	0	0	-
**Yellow coloration of skin and eyes**	0	0	-	0	0	-	0	0	-	0	0	-
**Labour pains or contractions**	2 (0.2)	0	-	0	0	-	2 (2.5)	0	0.25	1 (1.3)	0	0.50
**Fetal movement (normal)**	823 (99.3)	833 (99.6)	0.49	0	0	-	79 (98.8)	78 (97.5)	0.88	56 (70)	61 (76.3)	0.67
**Fall, injury or trauma**	3 (0.4)	12 (1.4)	0.04	0	1 (1.3)	1.0	3 (3.8)	9 (11.3)	0.14	1 (1.3)	3 (3.8)	0.63
**Sought care from a medical professional for a health problem**	173 (20.9)	184 (22.0)	0.55	10 (12.5)	10 (12.5)	1.0	68 (85.0)	68 (85.0)	0.96	35 (43.8)	25 (31.3)	0.18
**Sought care from a non-medical professional for a health problem**	4 (0.5)	11 (1.3)	0.14	0	3 (3.8)	0.25	3 (3.8)	7 (8.8)	0.34	0	1 (1.3)	1.0
**Ever reported symptoms suggestive of possible hypercalcemia[7]**	446 (53.8)	441 (52.8)	0.71	54 (67.5)	50 (62.5)	0.51	75 (93.8)	71 (88.8)	0.68	48 (60.0)	43 (53.8)	0.56

[1] n (%); Proportions of total number of symptom events reported is calculated using the total number of events reported in the placebo group (829) and in the vitamin D group (836) as the denominators.

[2] n (%); Proportion of women reporting the symptom at baseline, at least once during follow-up, and at least once anytime after 60 days of follow-up; denominators were total number of women enrolled in the placebo group (n=80) and vitamin D group (n=80).

[3] p-value for total no. of reported events was estimated via generalized estimating equation (exchangeable correlation, robust standard error) to account for within-subject correlation of repeated events. None of the pair-wise comparisons were statistically significant after correction for multiplicity using the Holm method (adjusted critical *P* value of 0.0018).

[4] None of the pair-wise comparisons were statistically significant after correction for multiplicity using the Holm method (adjusted critical *P* value of 0.0019)

[5] None of the pair-wise comparisons were statistically significant after correction for multiplicity using the Holm method (adjusted critical P value of 0.0015)

[6] None of the pair-wise comparisons were statistically significant after correction for multiplicity using the Holm method (adjusted critical P value of 0.0017)

[7] Symptoms suggestive of possible hypercalcemia included: decreased appetite, vomiting, fever, constipation, abdominal pain, excessive thirst, frequent urination, muscle weakness, back pain or cramps, leg pain or cramps, arm pain or cramps, mental confusion, and depressed mood.

Table S3: Biochemical adverse events at any time during follow-up

ID	Group	Event			Biochemistry			Comment
		Description	Time of onset		Albumin – adjusted serum [Ca] (mmol/L)	Urine Ca:Cr (mmol/ mmol)	25(OH)D (nmol/L)	
42	Vit D	Transient hypercalcemia (albumin adjusted serum Ca >2.60)	At delivery, 37 weeks gestation; day 55 of follow-up	*Baseline*	2.29	0.22	22	Repeat values were normal. No associated clinical concerns.
				Delivery	2.62	0.02	118	
				Repeat test (Day 58)	2.45	–	–	
				Repeat test (Day 67)	2.45	–	–	
				Range	2.29 – 2.62	0.02 – 0.22	22 – 118	
63	Vit D	Persistent hypercalciuria (2 consecutive results of Calcium:Creatinine ratio >1.0)	At 29 weeks gestation; day 14 of follow-up	*Baseline*	2.32	0.68	54	Repeat values were normal. No associated clinical concerns. Maternal status improved within 3 days. Renal ultrasound normal.
				Day 14	–	1.08	–	
				Repeat test (Day 16)	2.50	1.11	115	
				Delivery	2.42	0.23	147	
				Range	2.32 – 2.55	0.23 – 1.11	54 – 147	
13	Placebo	Calcium:Creatinine ratio >1.0	At delivery, 38 weeks gestation; day 68 of follow-up	*Baseline*	2.41	0.30	39	Repeat values were normal. No associated clinical concerns.
				Delivery	2.46	1.2	49	
				Repeat test (Day 70)	–	0.28	–	
				Range	2.35 – 2.46	0.18 – 1.2	39 – 49	
77	Vit. D	Calcium:Creatinine ratio >1.0	At delivery, 31 weeks gestation; day 30 of follow-up	*Baseline*	2.38	0.62	85	Repeat values were normal. No associated clinical concerns.
				Delivery	2.59	1.95	100	
				Repeat test (Day 34)	–	0.58	–	
				Range	2.38 – 2.59	0.26 – 1.95	85 – 116	
79	Placebo	Calcium:Creatinine ratio >1.0	At 29 weeks gestation; day 14 of follow-up	*Baseline*	2.47	0.68	38	Repeat values were normal. No associated clinical concerns.
				Day 14	–	1.11	–	
				Repeat test (Day 16)	–	0.26	–	
				Delivery	2.36	0.10	28	
				Range	2.36 – 2.49	0.10 – 1.11	19 – 38	
93	Placebo	Calcium:Creatinine ratio >1.0	At delivery, 38 weeks gestation; day 82 of follow-up	*Baseline*	2.21	0.22	31	Repeat values were normal. No associated clinical concerns.
				Delivery	2.44	1.26	45	
				Repeat test (Day 86)	–	0.74	–	
				Range	2.21 – 2.44	0.22 – 1.26	20 – 45	
111	Vit. D	Calcium:Creatinine ratio >1.0	At 29 weeks gestation; day 14 of follow-up	*Baseline*	2.35	0.64	48	Repeat values were normal. No associated clinical concerns.
				Day 14	–	1.25	–	
				Repeat test (Day 17)	–	0.15	–	
				Range	2.35 – 2.37	0.09 – 1.25	48 – 170	
152	Vit. D	Calcium:Creatinine ratio >1.0	At delivery, 39 weeks gestation; day 95 of follow-up	*Baseline*	2.42	0.88	82	Repeat values were normal. No associated clinical concerns..
				Delivery	2.42	2.26	136	
				Repeat test (Day 97)	–	0.71	–	
				Range	2.42 – 2.58	0.28 – 2.26	82 – 136	
3	Placebo	Calcium:Creatinine ratio >0.8 and >2-fold difference from baseline	At 30 weeks gestation; day 14 of follow-up	*Baseline*	2.40	0.24	56	Repeat values were normal. No associated clinical concerns.
				Day 14	–	0.94	–	
				Repeat test (Day 17)	–	0.82	–	
				Range	2.34 – 2.40	0.24 – 0.94	53 – 56	

ID	Group	Event		Biochemistry				Comment
		Description	Time of onset		Albumin – adjusted serum [Ca] (mmol/L)	Urine Ca:Cr (mmol/ mmol)	25(OH)D (nmol/L)	
17	Placebo	Calcium:Creatinine ratio >0.8 and >2-fold difference from baseline	At delivery, 38 weeks gestation; day 78 of follow-up	*Baseline*	2.39	0.11	80	Repeat values were normal. No associated clinical concerns.
				Delivery	2.43	0.87	74	
				Repeat test (Day 79)	–	0.42	–	
				Range	2.39 – 2.43	0.11 – 0.87	74 - 80	
83	Placebo	Calcium:Creatinine ratio >0.8 and >2-fold difference from baseline.	At 30 weeks gestation; day 14 of follow-up	*Baseline*	2.38	0.25	44	Repeat values were normal. No associated clinical concerns.
				Day 14	–	0.82	–	
				Repeat test (Day 15)	–	0.25	–	
				Range	–	0.25 – 0.82	–	
82	Vit. D	Calcium:Creatinine ratio >0.8 and >2-fold difference from baseline.	At 28 weeks gestation, day 14 of follow-up	*Baseline*	2.35	0.08	60	No associated clinical concerns. Calcium:creatinine ratio returned to normal by delivery.
				Day 14	–	0.84	–	
				Delivery (Day 105)	2.42	0.26	129	
				Range	2.35 – 2.42	0.08 – 0.84	60 – 129	
96	Vit. D	Calcium:Creatinine ratio >0.8 and >2-fold difference from baseline.	At delivery, 37 weeks gestation; day 74 of follow-up	*Baseline*	2.30	0.38	56	No associated clinical concerns. Normal serum albumin-adjusted calcium on day of event (2.43 mmol/L).
				Delivery	2.43	0.85	137	
				Repeat test	–	–	–	
				Range	2.30 – 2.45	0.38 – 0.85	56 – 137	
122	Vit. D	Calcium:Creatinine ratio >0.8 and >2-fold difference from baseline	At 31 weeks gestation; day 14 of follow-up	*Baseline*	2.39	0.26	15	No associated clinical concerns. Normal serum albumin-adjusted calcium within same week as event (2.34 mmol/L)
				Day 14	–	0.93	–	
				Delivery (Day 70)	2.41	0.15	78	
				Range	2.39 – 2.41	0.15 – 0.93	15 – 78	
136	Vit. D	Calcium:Creatinine ratio >0.8 and >2-fold difference from baseline	At 29 weeks gestation; day 14 of follow-up	*Baseline*	2.28	0.32	39	Repeat values were normal. No associated clinical concerns. Renal ultrasound normal. Maternal status improved.
				Day 14	–	0.87	–	
				Repeat test (Day 18)	–	0.86	–	
				Range	2.28 – 2.28	0.32 – 0.86	39 – 99	
142	Vit. D	Calcium:Creatinine ratio >0.8 and >2-fold difference from baseline	At delivery, 36 weeks gestation; day 70 of follow-up	*Baseline*	2.22	0.19	19	Repeat values were normal. No associated clinical concerns.
				Delivery	2.50	0.96	147	
				Repeat test (Day 73)	–	0.04	–	
				Range	2.22 – 2.50	0.04 – 0.96	19 – 147	

Table S4: Biochemical and clinical data among participants with a clinical serious adverse event at any time during follow-up

ID	Group	Event: Description	Event: Time of onset		Biochemistry: Albumin – adjusted serum [Ca] (mmol/L)	Biochemistry: Urine Ca:Cr (mmol/mmol)	Biochemistry: [25(OH)D] (nmol/L)	Action	Outcome
4	Vit D	Intrauterine death	39 weeks gestation; day 69 of follow-up	*Baseline*<br>*Delivery*<br>*Range*<br>*Cord blood*	2.36<br>2.48<br>2.36 – 2.48<br>–	0.58<br>0.71<br>0.58 – 0.71<br>–	76<br>123<br>76 – 146<br>–	Assessed by tertiary-care hospital.	Discharged without complications after obstetric management.
13	Placebo	Intrauterine death	38 weeks gestation; day 68 of follow-up	*Baseline*<br>*Delivery*<br>*Range*<br>*Cord blood*	2.41<br>2.46<br>2.35– 2.46<br>–	0.30<br>1.20<br>0.30 – 1.20<br>–	39<br>49<br>39 – 49<br>–	Assessed by tertiary-care hospital.	Discharged without complications after obstetric management.
42	VitD	Gastroenteritis	Delivery, at 37 weeks gestation; day 55 of follow-up.	*Baseline*<br>*Event*<br>*Post-event*<br>*Range*<br>*Cord blood*	2.29<br>2.62<br>2.45<br>2.29 – 2.62<br>3.0	0.22<br>0.02<br>–<br>0.02 – 0.22<br>–	22<br>118<br>101<br>22 – 118<br>115	Admitted to tertiary-care hospital	Discharged on day 2 of admission. Delivered live born term infant. No further maternal or neonatal complications.
77	VitD	Preterm delivery	Delivery, at 31 weeks gestation; day 30 of follow-up.	*Baseline*<br>*Event*<br>*Post-event*<br>*Range*<br>*Cord blood*	2.38<br>2.46<br>–<br>2.38 – 2.59<br>–	0.62<br>0.18<br>–<br>0.18 – 1.95<br>–	85<br>100<br>–<br>85 – 116<br>–	Admitted to tertiary-care hospital.	Discharged on day 8 of admission; delivered pre-term, low birthweight infant.
68	Placebo	Injury (15% flame burn)	38 weeks gestation; day 87 of follow-up.	*Baseline*<br>*Event*<br>*Post-event*<br>*Range*<br>*Cord blood*	2.30<br>2.31<br>2.46<br>2.31 – 2.46<br>2.44	0.14<br>0.04<br>0.05<br>0.04 – 0.14<br>–	26<br>9<br>–<br>9 – 26<br>13	Admitted to tertiary-care hospital.	Delivered term infant by uncomplicated vaginal delivery. No further maternal or infant complications.
155	Placebo	Pre-eclampsia with twin pregnancy	Delivery, at 36 weeks gestation; day 70 of follow-up.	*Baseline*<br>*Event*<br>*Post-event*<br>*Range*<br>*Cord blood*	2.39<br>2.41<br>–<br>2.39 – 2.41<br>2.59	0.14<br>0.05<br>–<br>0.05 – 0.14<br>–	21<br>42<br>–<br>21 – 70<br>35	Admitted to tertiary-care hospital. Newborns delivered by cesarean section.	Discharged on day 12 of admission. Delivered healthy infants. No further maternal or infant complications.

Table S5: Biochemical and clinical data among newborns with a clinical serious adverse event (birth to 1 month postnatal).

ID	Group	Event		Biochemistry				Action	Outcome
		Description	Time of onset		Albumin – adjusted serum [Ca] (mmol/L)	Urine Ca:Cr (mmol/ mmol)	[25(OH)D] (nmol/L)		
22	Placebo	Hypoxic-ischemic encephalopathy (mild)	42 weeks gestation; day 114 of follow-up	*Mat. Baseline*	2.27	0.01	47	Admitted to tertiary-care hospital	Discharged on day 3 of admission. No further acute neonatal complications.
				Mat. Delivery	2.14	0.01	21		
				Mat. Range	2.14 – 2.27	0.01 – 0.26	47 – 21		
				Cord blood	2.49	–	5		
				Infant (4 days)	2.14	–	–		
32	Placebo	Meconium aspiration syndrome	Delivery, at 43 weeks gestation; day 105 of follow-up.	*Mat. Baseline*	2.51	0.62	65	Admitted to tertiary-care hospital	Discharged on day 4 of admission. No further acute neonatal complications.
				Mat. Delivery	2.55	0.15	31		
				Mat. Range	2.45 – 2.55	0.15 – 0.62	31 – 67		
				Cord blood	2.73	–	43		
39	Placebo	Preterm; very low birth weight	At delivery, 31 weeks gestation; day 11 of follow-up	*Mat. Baseline*	2.42	0.49	50	Admitted to tertiary-care hospital.	Infant reported to have died within 5 minutes of birth in the home. No known maternal complications.
				Mat. Delivery	2.37	0.10	47		
				Mat. Range	2.37 – 2.42	0.10 – 0.49	47 – 50		
				Cord blood	–	–	–		
41	Vit D	Preterm; very low birth weight (880g); jaundice	At delivery, 31 weeks gestation; day 8 of follow-up	*Mat. Baseline*	2.33	0.01	71	Admitted to tertiary-care hospital.	Discharged on day 4 of admission. No further acute neonatal complications.
				Mat. Delivery	2.23	0.10	114		
				Mat. Range	2.23 – 2.33	0.01 – 0.10	71 – 114		
				Cord blood	–	–	–		
46	Placebo	Hypoxic-ischemic encephalopathy (severe); neonatal seizures; sepsis	At delivery, at 39 weeks gestation; day 79 of follow-up.	*Mat. Baseline*	2.41	0.17	61	Admitted to tertiary-care hospital.	Neonatal death on day 10 of hospitalization due to sepsis and multi-organ failure.
				Mat. Delivery	2.46	0.06	56		
				Mat. Range	2.39 – 2.46	0.06 – 0.39	52 – 61		
				Cord blood	2.7	–	42		
84	Placebo	Hypoxic-ischemic encephalopathy (severe)	At delivery, 39 weeks gestation; day 126 of follow-up	*Mat. Baseline*	2.31	0.47	38	Admitted to tertiary-care hospital.	Neonatal death on day 7 of hospitalization due to severe hypoxic-ischemic encephalopathyand respiratory failure.
				Mat. Delivery	2.40	0.17	32		
				Mat. Range	2.31 – 1.40	0.17 – 0.47	17 – 38		
				Cord blood	2.74	–	33		
88	Placebo	Sub-acute intestinal obstruction	Postnatal; Infant age 11 days at onset.	*Mat. Baseline*	2.39	0.13	12	Admitted to tertiary-care hospital.	Discharged on day 5 of admission. No further neonatal complications.
				Mat. Delivery	2.44	0.07	11		
				Mat. Range	2.39 – 2.44	0.04 – 0.13	11 – 16		
				Cord blood	2.80	–	38		

ID	Group	Event			Biochemistry			Action	Outcome
		Description	Time of onset		Albumin – adjusted serum [Ca] (mmol/L)	Urine Ca:Cr (mmol/ mmol)	[25(OH)D] (nmol/L)		
97	Vit D	Pneumonia	Postnatal; Infant age 16 days at onset.	*Mat. Baseline*	2.44	0.33	63	Admitted to tertiary-care hospital.	Treated and discharged on day 5 of admission. No further infant complications.
				Mat. Delivery	2.44	0.07	126		
				Mat. Range	2.44 – 2.47	0.07 – 0.33	63 – 126		
				Cord blood	2.85	–	83		
				Infant (18 days)	2.84	–	71		
117	Vit D	Cardio-respiratory failure; low birth weight	At delivery, 37 weeks gestation; day 62 of follow-up	*Mat. Baseline*	2.42	0.05	84	Assessed in a tertiary-care hospital	Neonatal death at 5 hours of life due to cardio-respiratory failure of unknown etiology.
				Mat. Delivery	2.44	0.02	174		
				Mat. Range	2.37 – 2.44	0.02 – 0.09	84 – 174		
				Cord blood	2.79	–	104		
